# Di-μ-chlorido-bis­{bis­[4-(adamantan-1-ylcarbamo­yl)pyridine-κ*N*]chlorido­copper(II)} hemihydrate

**DOI:** 10.1107/S1600536812000670

**Published:** 2012-01-14

**Authors:** Ying-Chun Wang

**Affiliations:** a College of Chemistry and Chemical Engineering, Southeast University, Nanjing 210096, People’s Republic of China

## Abstract

In the centrosymmetric dimeric title compound, [Cu_2_Cl_4_(C_16_H_20_N_2_O)_4_]·0.5H_2_O, the Cu^II^ atom is in a distorted trigonal–bipyramidal environment defined by two bridging Cl atoms, one terminal Cl atom and two N atoms from two monodentate 4-(adamantan-1-ylcarbamo­yl)pyridine ligands. The amine N atoms are involved in intra­molecular N—H⋯O and inter­molecular N—H⋯Cl hydrogen bonds. The latter hydrogen bonds link the complex mol­ecules into a ribbon along [010]. The uncoordinated water mol­ecule is 0.25-occupied.

## Related literature

For the structures of related amino compounds, see: Fu *et al.* (2007 ▶, 2008 ▶, 2009 ▶); Fu & Xiong (2008 ▶). For the ferroelectric properties of related amino derivatives, see: Fu *et al.* (2011*a* ▶,*b* ▶,*c* ▶).
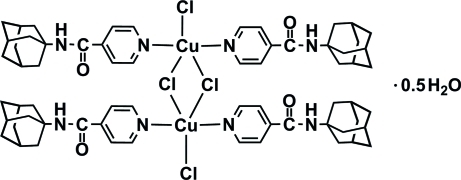


## Experimental

### 

#### Crystal data

[Cu_2_Cl_4_(C_16_H_20_N_2_O)_4_]·0.5H_2_O
*M* *_r_* = 1303.25
Triclinic, 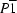

*a* = 6.739 (4) Å
*b* = 11.149 (6) Å
*c* = 21.814 (12) Å
α = 92.221 (6)°
β = 95.993 (8)°
γ = 96.727 (9)°
*V* = 1616.6 (16) Å^3^
*Z* = 1
Mo *K*α radiation
μ = 0.88 mm^−1^
*T* = 295 K
0.30 × 0.25 × 0.15 mm

#### Data collection

Rigaku Mercury2 CCD diffractometer
Absorption correction: multi-scan (*CrystalClear*; Rigaku, 2005 ▶) *T*_min_ = 0.779, *T*_max_ = 0.880
16110 measured reflections
6320 independent reflections
4988 reflections with *I* > 2σ(*I*)
*R*_int_ = 0.054

#### Refinement

*R*[*F* ^2^ > 2σ(*F* ^2^)] = 0.069
*wR*(*F* ^2^) = 0.197
*S* = 1.06
6320 reflections
379 parameters
H-atom parameters constrained
Δρ_max_ = 1.19 e Å^−3^
Δρ_min_ = −0.77 e Å^−3^

### 

Data collection: *CrystalClear* (Rigaku, 2005 ▶); cell refinement: *CrystalClear*; data reduction: *CrystalClear*; program(s) used to solve structure: *SHELXTL* (Sheldrick, 2008 ▶); program(s) used to refine structure: *SHELXTL*; molecular graphics: *XP* in *SHELXTL* and *DIAMOND* (Brandenburg, 1999 ▶); software used to prepare material for publication: *SHELXTL*.

## Supplementary Material

Crystal structure: contains datablock(s) I, global. DOI: 10.1107/S1600536812000670/hy2504sup1.cif

Structure factors: contains datablock(s) I. DOI: 10.1107/S1600536812000670/hy2504Isup2.hkl

Additional supplementary materials:  crystallographic information; 3D view; checkCIF report

## Figures and Tables

**Table 1:** Hydrogen-bond geometry (Å, °)

*D*—H⋯*A*	*D*—H	H⋯*A*	*D*⋯*A*	*D*—H⋯*A*
N1—H1*A*⋯O1^i^	0.86	2.35	2.969 (5)	129
N2—H2*A*⋯Cl1^ii^	0.86	2.66	3.499 (4)	165

Symmetry codes: (i) 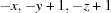; (ii) 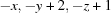; (iii) 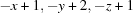.

**Fig. 1.**
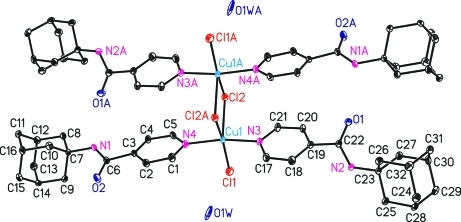
Molecular structure of the title compound. Displacement ellipsoids are drawn at the 30% probability level. H atoms have been omitted for clarity. [Symmetry code: (A) -x, 1-y, 1-z.]

**Fig. 2.**
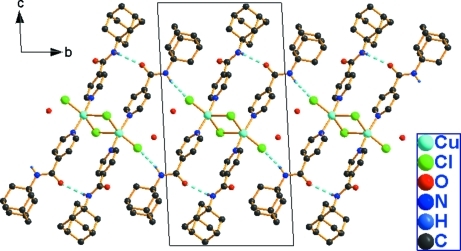
The crystal packing of the title compound viewed along the a axis, showing the hydrogen bonded ribbon. H atoms not involved in hydrogen bonds (dashed line) have been omitted for clarity.

## supplementary crystallographic information

### Comment

Amino compounds have attracted more attention as phase transition dielectric materials for their applications in memory storage (Fu *et al.*, 2007, 2008, 2009; Fu & Xiong, 2008). With the purpose of obtaining phase transition crystals of amino compounds, various amines have been studied and a series of new materials with this organic molecules have been elaborated (Fu *et al.*, 2011*a*,b,c). In this study, we describe the crystal structure of the title compound.

The asymmetric unit is composed of two 4-[(1-adamantyl)carbamoyl]pyridine ligands, two Cl^-^ anions, one Cu^II^ ion and a quarter of water molecule (Fig. 1). The two pyridine rings are twisted from each other by a dihedral angle of 11.14 (1)°. The dimeric complex molecule is centrosymmetric. The distorted trigonal-bipyramidal environment around the Cu^II^ ion is defined by two bridging Cl atoms, one terminal Cl atom and two N atoms from two monodentate organic ligands. The geometric parameters in the title compound are in a normal range.

In the crystal, the amino N atoms are involved in an intramolecular N—H···O hydrogen bond and an intermolecular N—H···Cl hydrogen bond. These hydrogen bonds link the complex molecules into a one-dimensional ribbon along [0 1 0] (Table 1 and Fig. 2).

### Experimental

CuCl_2_.6H_2_O (2 mmol) and 4-[(1-adamantyl)carbamoyl]pyridine (2 mmol) were dissolved in 70% aqueous methanol solution, and then 2 ml HCl was added. Single crystals suitable for X-ray diffraction analysis were obtained from slow evaporation of the solution at room temperature after two weeks.

### Refinement

H atoms attached to C and N atoms were positioned geometrically and treated as riding, with C—H = 0.93 (aromatic), 0.97 (methylene) and 0.98 Å (methine) and N—H = 0.86 Å and with *U*_iso_(H) = 1.2*U*_eq_(C, N). H atoms bonded to O atom were located in a difference Fourier map and restrained with H—O = 0.82 (1) Å. In the last stage of refinement, they were treated as riding atoms with *U*_iso_(H) = 1.5*U*_eq_(O). The highest residual electron density was found at 0.97 Å from Cl1 atom and the deepest hole at 0.80 Å from Cl1 atom.

### Crystal data

**Table:**

[Cu_2_Cl_4_(C_16_H_20_N_2_O)_4_]·0.5H_2_O	*Z* = 1
*M**_r_* = 1303.25	*F*(000) = 683
Triclinic, *P*1	*D*_x_ = 1.339 Mg m^−^^3^
Hall symbol: -P 1	Mo *K*α radiation, λ = 0.71073 Å
*a* = 6.739 (4) Å	Cell parameters from 4355 reflections
*b* = 11.149 (6) Å	θ = 2.6–27.5°
*c* = 21.814 (12) Å	µ = 0.88 mm^−^^1^
α = 92.221 (6)°	*T* = 295 K
β = 95.993 (8)°	Block, colorless
γ = 96.727 (9)°	0.30 × 0.25 × 0.15 mm
*V* = 1616.6 (16) Å^3^	

### Data collection

**Table:**

Rigaku Mercury2 CCD diffractometer	6320 independent reflections
Radiation source: fine-focus sealed tube	4988 reflections with *I* > 2σ(*I*)
graphite	*R*_int_ = 0.054
Detector resolution: 13.6612 pixels mm^-1^	θ_max_ = 26.0°, θ_min_ = 1.9°
ω scans	*h* = −8→8
Absorption correction: multi-scan (*CrystalClear*; Rigaku, 2005)	*k* = −13→13
*T*_min_ = 0.779, *T*_max_ = 0.880	*l* = −26→26
16110 measured reflections	

### Refinement

**Table:**

Refinement on *F*^2^	Primary atom site location: structure-invariant direct methods
Least-squares matrix: full	Secondary atom site location: difference Fourier map
*R*[*F*^2^ > 2σ(*F*^2^)] = 0.069	Hydrogen site location: inferred from neighbouring sites
*wR*(*F*^2^) = 0.197	H-atom parameters constrained
*S* = 1.06	*w* = 1/[σ^2^(*F*_o_^2^) + (0.1016*P*)^2^ + 0.817*P*] where *P* = (*F*_o_^2^ + 2*F*_c_^2^)/3
6320 reflections	(Δ/σ)_max_ < 0.001
379 parameters	Δρ_max_ = 1.19 e Å^−^^3^
0 restraints	Δρ_min_ = −0.77 e Å^−^^3^

### Special details

**Table:**

Geometry. All esds (except the esd in the dihedral angle between two l.s. planes) are estimated using the full covariance matrix. The cell esds are taken into account individually in the estimation of esds in distances, angles and torsion angles; correlations between esds in cell parameters are only used when they are defined by crystal symmetry. An approximate (isotropic) treatment of cell esds is used for estimating esds involving l.s. planes.
Refinement. Refinement of F^2^ against ALL reflections. The weighted R-factor wR and goodness of fit S are based on F^2^, conventional R-factors R are based on F, with F set to zero for negative F^2^. The threshold expression of F^2^ > 2sigma(F^2^) is used only for calculating R-factors(gt) etc. and is not relevant to the choice of reflections for refinement. R-factors based on F^2^ are statistically about twice as large as those based on F, and R- factors based on ALL data will be even larger.

### Fractional atomic coordinates and isotropic or equivalent isotropic displacement parameters (Å^2^)

**Table:**

	*x*	*y*	*z*	*U*_iso_*/*U*_eq_	Occ. (<1)
Cu1	0.02209 (9)	0.63814 (5)	0.46299 (2)	0.0322 (2)	
Cl1	−0.0961 (3)	0.78238 (13)	0.40137 (6)	0.0589 (4)	
Cl2	−0.24643 (16)	0.45318 (10)	0.46545 (5)	0.0337 (3)	
O1	−0.2052 (7)	0.8512 (3)	0.74349 (17)	0.0636 (12)	
O2	0.6396 (5)	0.5226 (3)	0.24014 (16)	0.0496 (9)	
N1	0.3965 (6)	0.3694 (3)	0.20396 (17)	0.0331 (9)	
H1A	0.2815	0.3324	0.2100	0.040*	
N2	−0.1049 (7)	1.0381 (3)	0.71060 (17)	0.0387 (10)	
H2A	−0.0776	1.0767	0.6785	0.046*	
N3	−0.0537 (6)	0.7312 (3)	0.53575 (17)	0.0366 (9)	
N4	0.1535 (6)	0.5718 (3)	0.39231 (16)	0.0334 (8)	
C1	0.3484 (7)	0.6058 (5)	0.3907 (2)	0.0396 (11)	
H1B	0.4175	0.6554	0.4232	0.048*	
C2	0.4541 (7)	0.5711 (4)	0.3431 (2)	0.0361 (11)	
H2B	0.5898	0.5987	0.3432	0.043*	
C3	0.3534 (7)	0.4941 (4)	0.29515 (19)	0.0282 (9)	
C4	0.1504 (7)	0.4574 (4)	0.29694 (19)	0.0325 (10)	
H4A	0.0793	0.4052	0.2658	0.039*	
C5	0.0534 (7)	0.4988 (4)	0.34532 (19)	0.0321 (10)	
H5A	−0.0838	0.4758	0.3454	0.038*	
C6	0.4783 (7)	0.4621 (4)	0.2442 (2)	0.0321 (10)	
C7	0.4921 (6)	0.3277 (4)	0.15020 (18)	0.0259 (9)	
C8	0.3411 (6)	0.2306 (4)	0.1135 (2)	0.0307 (10)	
H8A	0.2182	0.2645	0.1006	0.037*	
H8B	0.3085	0.1640	0.1395	0.037*	
C9	0.5378 (7)	0.4319 (4)	0.1079 (2)	0.0347 (10)	
H9A	0.4147	0.4651	0.0944	0.042*	
H9B	0.6302	0.4957	0.1305	0.042*	
C10	0.6852 (7)	0.2739 (5)	0.1707 (2)	0.0373 (11)	
H10A	0.6559	0.2074	0.1970	0.045*	
H10B	0.7807	0.3349	0.1942	0.045*	
C11	0.6269 (8)	0.1311 (4)	0.0778 (2)	0.0441 (13)	
H11A	0.5975	0.0639	0.1037	0.053*	
H11B	0.6850	0.1012	0.0422	0.053*	
C12	0.4312 (7)	0.1841 (4)	0.0564 (2)	0.0391 (11)	
H12A	0.3350	0.1212	0.0335	0.047*	
C13	0.4807 (8)	0.2882 (4)	0.0147 (2)	0.0411 (11)	
H13A	0.3589	0.3220	0.0004	0.049*	
H13B	0.5385	0.2587	−0.0210	0.049*	
C14	0.6310 (7)	0.3860 (4)	0.0516 (2)	0.0328 (10)	
H14A	0.6630	0.4534	0.0254	0.039*	
C15	0.8239 (7)	0.3321 (4)	0.0733 (2)	0.0403 (11)	
H15A	0.8838	0.3034	0.0378	0.048*	
H15B	0.9199	0.3938	0.0961	0.048*	
C16	0.7755 (7)	0.2287 (5)	0.1139 (2)	0.0414 (12)	
H16A	0.8994	0.1945	0.1273	0.050*	
C17	0.0825 (9)	0.8159 (5)	0.5631 (3)	0.0540 (15)	
H17A	0.2040	0.8309	0.5465	0.065*	
C18	0.0550 (9)	0.8840 (5)	0.6154 (2)	0.0527 (14)	
H18A	0.1539	0.9448	0.6324	0.063*	
C19	−0.1204 (8)	0.8601 (4)	0.6415 (2)	0.0392 (11)	
C20	−0.2667 (8)	0.7724 (4)	0.6124 (2)	0.0401 (11)	
H20A	−0.3891	0.7557	0.6284	0.048*	
C21	−0.2304 (7)	0.7102 (4)	0.5598 (2)	0.0394 (11)	
H21A	−0.3299	0.6522	0.5404	0.047*	
C22	−0.1500 (9)	0.9173 (4)	0.7036 (2)	0.0435 (12)	
C23	−0.0991 (7)	1.1090 (4)	0.76943 (19)	0.0312 (10)	
C24	−0.3064 (7)	1.0948 (4)	0.7938 (2)	0.0378 (11)	
H24A	−0.4059	1.1214	0.7636	0.045*	
H24B	−0.3466	1.0104	0.8007	0.045*	
C25	−0.0430 (8)	1.2429 (4)	0.7573 (2)	0.0371 (11)	
H25A	−0.1427	1.2686	0.7269	0.045*	
H25B	0.0862	1.2536	0.7411	0.045*	
C26	0.0598 (7)	1.0685 (4)	0.8172 (2)	0.0362 (10)	
H26A	0.1899	1.0776	0.8015	0.043*	
H26B	0.0251	0.9840	0.8249	0.043*	
C27	0.1282 (8)	1.2794 (4)	0.8652 (2)	0.0430 (12)	
H27A	0.2582	1.2893	0.8494	0.052*	
H27B	0.1380	1.3287	0.9033	0.052*	
C28	−0.0326 (8)	1.3205 (4)	0.8176 (2)	0.0383 (11)	
H28A	0.0036	1.4058	0.8097	0.046*	
C29	−0.2370 (8)	1.3047 (4)	0.8434 (2)	0.0456 (13)	
H29A	−0.3384	1.3326	0.8143	0.055*	
H29B	−0.2292	1.3527	0.8818	0.055*	
C30	−0.2954 (8)	1.1707 (5)	0.8546 (2)	0.0437 (12)	
H30A	−0.4264	1.1604	0.8707	0.052*	
C31	−0.1339 (9)	1.1290 (4)	0.9021 (2)	0.0471 (13)	
H31A	−0.1282	1.1756	0.9409	0.057*	
H31B	−0.1692	1.0444	0.9096	0.057*	
C32	0.0700 (8)	1.1463 (4)	0.8777 (2)	0.0413 (12)	
H32A	0.1715	1.1203	0.9082	0.050*	
O1W	0.500 (3)	0.9159 (17)	0.4447 (12)	0.125 (11)	0.25
H1WA	0.5145	0.9475	0.4800	0.187*	0.25
H1WB	0.3799	0.8940	0.4340	0.187*	0.25

### Atomic displacement parameters (Å^2^)

**Table:**

	*U*^11^	*U*^22^	*U*^33^	*U*^12^	*U*^13^	*U*^23^
Cu1	0.0445 (4)	0.0306 (3)	0.0236 (3)	0.0077 (2)	0.0115 (2)	−0.0042 (2)
Cl1	0.0939 (12)	0.0497 (8)	0.0404 (8)	0.0268 (8)	0.0188 (7)	0.0097 (6)
Cl2	0.0341 (6)	0.0375 (6)	0.0288 (6)	0.0025 (5)	0.0040 (4)	−0.0028 (4)
O1	0.123 (4)	0.0302 (19)	0.039 (2)	−0.006 (2)	0.039 (2)	−0.0058 (16)
O2	0.044 (2)	0.055 (2)	0.046 (2)	−0.0154 (17)	0.0200 (16)	−0.0178 (17)
N1	0.036 (2)	0.032 (2)	0.031 (2)	−0.0051 (16)	0.0170 (16)	−0.0065 (16)
N2	0.068 (3)	0.026 (2)	0.023 (2)	−0.0004 (19)	0.0162 (18)	−0.0035 (15)
N3	0.049 (2)	0.031 (2)	0.031 (2)	0.0095 (18)	0.0103 (18)	−0.0014 (16)
N4	0.040 (2)	0.035 (2)	0.026 (2)	0.0097 (17)	0.0078 (16)	−0.0021 (15)
C1	0.040 (3)	0.049 (3)	0.028 (2)	0.002 (2)	0.006 (2)	−0.010 (2)
C2	0.036 (2)	0.043 (3)	0.028 (2)	−0.004 (2)	0.0075 (19)	−0.0079 (19)
C3	0.037 (2)	0.027 (2)	0.022 (2)	0.0064 (18)	0.0099 (17)	0.0005 (17)
C4	0.035 (2)	0.037 (3)	0.025 (2)	0.002 (2)	0.0053 (18)	−0.0101 (18)
C5	0.031 (2)	0.042 (3)	0.024 (2)	0.000 (2)	0.0089 (17)	−0.0023 (18)
C6	0.038 (2)	0.035 (2)	0.024 (2)	0.002 (2)	0.0110 (18)	−0.0039 (18)
C7	0.030 (2)	0.027 (2)	0.022 (2)	0.0042 (18)	0.0085 (16)	−0.0023 (16)
C8	0.029 (2)	0.033 (2)	0.031 (2)	0.0011 (19)	0.0078 (18)	−0.0048 (18)
C9	0.045 (3)	0.028 (2)	0.034 (3)	0.010 (2)	0.012 (2)	0.0000 (19)
C10	0.034 (2)	0.048 (3)	0.032 (3)	0.011 (2)	0.0015 (19)	0.006 (2)
C11	0.060 (3)	0.028 (2)	0.050 (3)	0.013 (2)	0.027 (3)	0.002 (2)
C12	0.045 (3)	0.036 (3)	0.035 (3)	−0.003 (2)	0.011 (2)	−0.011 (2)
C13	0.053 (3)	0.045 (3)	0.026 (2)	0.008 (2)	0.008 (2)	−0.003 (2)
C14	0.045 (3)	0.028 (2)	0.028 (2)	0.005 (2)	0.014 (2)	0.0065 (18)
C15	0.035 (3)	0.044 (3)	0.045 (3)	0.006 (2)	0.018 (2)	0.002 (2)
C16	0.031 (2)	0.048 (3)	0.052 (3)	0.023 (2)	0.016 (2)	0.014 (2)
C17	0.064 (4)	0.042 (3)	0.058 (4)	−0.006 (3)	0.034 (3)	−0.014 (3)
C18	0.063 (3)	0.043 (3)	0.049 (3)	−0.012 (3)	0.021 (3)	−0.019 (2)
C19	0.063 (3)	0.028 (2)	0.029 (2)	0.006 (2)	0.017 (2)	−0.0049 (19)
C20	0.054 (3)	0.038 (3)	0.031 (3)	0.008 (2)	0.018 (2)	−0.010 (2)
C21	0.041 (3)	0.040 (3)	0.035 (3)	0.008 (2)	−0.001 (2)	−0.008 (2)
C22	0.073 (4)	0.026 (2)	0.032 (3)	0.000 (2)	0.020 (2)	−0.007 (2)
C23	0.045 (3)	0.024 (2)	0.025 (2)	0.0017 (19)	0.0082 (19)	−0.0059 (17)
C24	0.042 (3)	0.035 (3)	0.035 (3)	0.000 (2)	0.008 (2)	−0.009 (2)
C25	0.058 (3)	0.027 (2)	0.027 (2)	0.006 (2)	0.007 (2)	−0.0005 (18)
C26	0.045 (3)	0.029 (2)	0.037 (3)	0.013 (2)	0.008 (2)	0.0047 (19)
C27	0.052 (3)	0.036 (3)	0.037 (3)	−0.002 (2)	0.002 (2)	−0.008 (2)
C28	0.061 (3)	0.022 (2)	0.032 (3)	0.002 (2)	0.009 (2)	−0.0041 (18)
C29	0.058 (3)	0.031 (3)	0.051 (3)	0.013 (2)	0.013 (3)	−0.010 (2)
C30	0.045 (3)	0.045 (3)	0.043 (3)	0.003 (2)	0.023 (2)	−0.012 (2)
C31	0.082 (4)	0.034 (3)	0.026 (3)	0.003 (3)	0.015 (2)	−0.004 (2)
C32	0.050 (3)	0.038 (3)	0.034 (3)	0.009 (2)	−0.005 (2)	0.000 (2)
O1W	0.074 (13)	0.072 (13)	0.23 (3)	−0.003 (11)	0.064 (16)	−0.097 (16)

### Geometric parameters (Å, °)

**Table:**

Cu1—N3	2.006 (4)	C13—H13B	0.9700
Cu1—N4	2.015 (4)	C14—C15	1.533 (7)
Cu1—Cl1	2.2961 (16)	C14—H14A	0.9800
Cu1—Cl2^i^	2.3978 (15)	C15—C16	1.507 (7)
Cu1—Cl2	2.5854 (16)	C15—H15A	0.9700
O1—C22	1.222 (6)	C15—H15B	0.9700
O2—C6	1.223 (5)	C16—H16A	0.9800
N1—C6	1.355 (6)	C17—C18	1.387 (7)
N1—C7	1.480 (5)	C17—H17A	0.9300
N1—H1A	0.8600	C18—C19	1.368 (7)
N2—C22	1.344 (6)	C18—H18A	0.9300
N2—C23	1.476 (5)	C19—C20	1.389 (7)
N2—H2A	0.8600	C19—C22	1.516 (6)
N3—C17	1.315 (7)	C20—C21	1.375 (6)
N3—C21	1.350 (6)	C20—H20A	0.9300
N4—C1	1.328 (6)	C21—H21A	0.9300
N4—C5	1.353 (6)	C23—C26	1.534 (6)
C1—C2	1.387 (6)	C23—C25	1.536 (6)
C1—H1B	0.9300	C23—C24	1.539 (6)
C2—C3	1.392 (6)	C24—C30	1.535 (6)
C2—H2B	0.9300	C24—H24A	0.9700
C3—C4	1.386 (6)	C24—H24B	0.9700
C3—C6	1.519 (5)	C25—C28	1.536 (6)
C4—C5	1.390 (6)	C25—H25A	0.9700
C4—H4A	0.9300	C25—H25B	0.9700
C5—H5A	0.9300	C26—C32	1.542 (6)
C7—C10	1.528 (6)	C26—H26A	0.9700
C7—C8	1.534 (6)	C26—H26B	0.9700
C7—C9	1.534 (6)	C27—C32	1.532 (7)
C8—C12	1.539 (6)	C27—C28	1.543 (7)
C8—H8A	0.9700	C27—H27A	0.9700
C8—H8B	0.9700	C27—H27B	0.9700
C9—C14	1.534 (6)	C28—C29	1.535 (7)
C9—H9A	0.9700	C28—H28A	0.9800
C9—H9B	0.9700	C29—C30	1.535 (7)
C10—C16	1.530 (6)	C29—H29A	0.9700
C10—H10A	0.9700	C29—H29B	0.9700
C10—H10B	0.9700	C30—C31	1.548 (8)
C11—C16	1.524 (7)	C30—H30A	0.9800
C11—C12	1.543 (7)	C31—C32	1.519 (7)
C11—H11A	0.9700	C31—H31A	0.9700
C11—H11B	0.9700	C31—H31B	0.9700
C12—C13	1.530 (7)	C32—H32A	0.9800
C12—H12A	0.9800	O1W—H1WA	0.8258
C13—C14	1.538 (7)	O1W—H1WB	0.8262
C13—H13A	0.9700		
			
N3—Cu1—N4	167.22 (17)	C16—C15—H15A	109.7
N3—Cu1—Cl1	88.13 (12)	C14—C15—H15A	109.7
N4—Cu1—Cl1	90.06 (12)	C16—C15—H15B	109.7
N3—Cu1—Cl2^i^	87.18 (12)	C14—C15—H15B	109.7
N4—Cu1—Cl2^i^	89.94 (12)	H15A—C15—H15B	108.2
Cl1—Cu1—Cl2^i^	158.66 (6)	C15—C16—C11	109.7 (4)
N3—Cu1—Cl2	96.71 (12)	C15—C16—C10	110.0 (4)
N4—Cu1—Cl2	95.73 (12)	C11—C16—C10	109.7 (4)
Cl1—Cu1—Cl2	111.32 (7)	C15—C16—H16A	109.1
Cl2^i^—Cu1—Cl2	89.91 (5)	C11—C16—H16A	109.1
Cu1^i^—Cl2—Cu1	90.09 (5)	C10—C16—H16A	109.1
C6—N1—C7	124.3 (4)	N3—C17—C18	123.6 (5)
C6—N1—H1A	117.9	N3—C17—H17A	118.2
C7—N1—H1A	117.8	C18—C17—H17A	118.2
C22—N2—C23	124.6 (4)	C19—C18—C17	118.8 (5)
C22—N2—H2A	117.7	C19—C18—H18A	120.6
C23—N2—H2A	117.7	C17—C18—H18A	120.6
C17—N3—C21	118.0 (4)	C18—C19—C20	118.0 (4)
C17—N3—Cu1	117.1 (3)	C18—C19—C22	122.0 (5)
C21—N3—Cu1	124.9 (3)	C20—C19—C22	119.7 (4)
C1—N4—C5	118.3 (4)	C21—C20—C19	119.8 (5)
C1—N4—Cu1	117.8 (3)	C21—C20—H20A	120.1
C5—N4—Cu1	123.9 (3)	C19—C20—H20A	120.1
N4—C1—C2	123.4 (4)	N3—C21—C20	121.6 (5)
N4—C1—H1B	118.3	N3—C21—H21A	119.2
C2—C1—H1B	118.3	C20—C21—H21A	119.2
C1—C2—C3	118.8 (4)	O1—C22—N2	125.3 (4)
C1—C2—H2B	120.6	O1—C22—C19	118.4 (4)
C3—C2—H2B	120.6	N2—C22—C19	116.2 (4)
C4—C3—C2	118.0 (4)	N2—C23—C26	109.8 (4)
C4—C3—C6	126.2 (4)	N2—C23—C25	108.0 (3)
C2—C3—C6	115.7 (4)	C26—C23—C25	109.5 (4)
C3—C4—C5	119.8 (4)	N2—C23—C24	110.9 (4)
C3—C4—H4A	120.1	C26—C23—C24	110.2 (4)
C5—C4—H4A	120.1	C25—C23—C24	108.4 (4)
N4—C5—C4	121.6 (4)	C30—C24—C23	109.5 (4)
N4—C5—H5A	119.2	C30—C24—H24A	109.8
C4—C5—H5A	119.2	C23—C24—H24A	109.8
O2—C6—N1	124.1 (4)	C30—C24—H24B	109.8
O2—C6—C3	119.4 (4)	C23—C24—H24B	109.8
N1—C6—C3	116.4 (4)	H24A—C24—H24B	108.2
N1—C7—C10	111.2 (4)	C23—C25—C28	109.9 (4)
N1—C7—C8	107.4 (3)	C23—C25—H25A	109.7
C10—C7—C8	109.5 (4)	C28—C25—H25A	109.7
N1—C7—C9	110.7 (3)	C23—C25—H25B	109.7
C10—C7—C9	109.8 (4)	C28—C25—H25B	109.7
C8—C7—C9	108.1 (4)	H25A—C25—H25B	108.2
C7—C8—C12	110.0 (3)	C23—C26—C32	109.4 (4)
C7—C8—H8A	109.7	C23—C26—H26A	109.8
C12—C8—H8A	109.7	C32—C26—H26A	109.8
C7—C8—H8B	109.7	C23—C26—H26B	109.8
C12—C8—H8B	109.7	C32—C26—H26B	109.8
H8A—C8—H8B	108.2	H26A—C26—H26B	108.2
C14—C9—C7	109.9 (3)	C32—C27—C28	109.3 (4)
C14—C9—H9A	109.7	C32—C27—H27A	109.8
C7—C9—H9A	109.7	C28—C27—H27A	109.8
C14—C9—H9B	109.7	C32—C27—H27B	109.8
C7—C9—H9B	109.7	C28—C27—H27B	109.8
H9A—C9—H9B	108.2	H27A—C27—H27B	108.3
C7—C10—C16	109.5 (4)	C29—C28—C25	110.0 (4)
C7—C10—H10A	109.8	C29—C28—C27	109.2 (4)
C16—C10—H10A	109.8	C25—C28—C27	109.0 (4)
C7—C10—H10B	109.8	C29—C28—H28A	109.6
C16—C10—H10B	109.8	C25—C28—H28A	109.6
H10A—C10—H10B	108.2	C27—C28—H28A	109.6
C16—C11—C12	109.6 (4)	C28—C29—C30	109.5 (4)
C16—C11—H11A	109.8	C28—C29—H29A	109.8
C12—C11—H11A	109.8	C30—C29—H29A	109.8
C16—C11—H11B	109.8	C28—C29—H29B	109.8
C12—C11—H11B	109.8	C30—C29—H29B	109.8
H11A—C11—H11B	108.2	H29A—C29—H29B	108.2
C13—C12—C8	110.1 (4)	C29—C30—C24	109.8 (4)
C13—C12—C11	108.8 (4)	C29—C30—C31	108.9 (4)
C8—C12—C11	108.9 (4)	C24—C30—C31	109.3 (4)
C13—C12—H12A	109.7	C29—C30—H30A	109.6
C8—C12—H12A	109.7	C24—C30—H30A	109.6
C11—C12—H12A	109.7	C31—C30—H30A	109.6
C12—C13—C14	108.9 (4)	C32—C31—C30	109.9 (4)
C12—C13—H13A	109.9	C32—C31—H31A	109.7
C14—C13—H13A	109.9	C30—C31—H31A	109.7
C12—C13—H13B	109.9	C32—C31—H31B	109.7
C14—C13—H13B	109.9	C30—C31—H31B	109.7
H13A—C13—H13B	108.3	H31A—C31—H31B	108.2
C9—C14—C15	109.4 (4)	C31—C32—C27	110.5 (4)
C9—C14—C13	109.4 (4)	C31—C32—C26	108.9 (4)
C15—C14—C13	109.4 (4)	C27—C32—C26	109.3 (4)
C9—C14—H14A	109.5	C31—C32—H32A	109.3
C15—C14—H14A	109.5	C27—C32—H32A	109.3
C13—C14—H14A	109.5	C26—C32—H32A	109.3
C16—C15—C14	109.7 (4)	H1WA—O1W—H1WB	109.6
			
N3—Cu1—Cl2—Cu1^i^	−87.15 (12)	C12—C13—C14—C9	−59.6 (5)
N4—Cu1—Cl2—Cu1^i^	89.93 (12)	C12—C13—C14—C15	60.3 (5)
Cl1—Cu1—Cl2—Cu1^i^	−177.76 (5)	C9—C14—C15—C16	59.7 (5)
Cl2^i^—Cu1—Cl2—Cu1^i^	0.000 (2)	C13—C14—C15—C16	−60.2 (5)
N4—Cu1—N3—C17	−7.0 (9)	C14—C15—C16—C11	60.1 (5)
Cl1—Cu1—N3—C17	−89.0 (4)	C14—C15—C16—C10	−60.6 (5)
Cl2^i^—Cu1—N3—C17	70.1 (4)	C12—C11—C16—C15	−60.4 (5)
Cl2—Cu1—N3—C17	159.7 (4)	C12—C11—C16—C10	60.5 (5)
N4—Cu1—N3—C21	175.6 (6)	C7—C10—C16—C15	60.2 (5)
Cl1—Cu1—N3—C21	93.6 (4)	C7—C10—C16—C11	−60.6 (5)
Cl2^i^—Cu1—N3—C21	−107.2 (4)	C21—N3—C17—C18	0.4 (8)
Cl2—Cu1—N3—C21	−17.7 (4)	Cu1—N3—C17—C18	−177.2 (5)
N3—Cu1—N4—C1	16.7 (9)	N3—C17—C18—C19	2.1 (9)
Cl1—Cu1—N4—C1	98.5 (4)	C17—C18—C19—C20	−3.2 (8)
Cl2^i^—Cu1—N4—C1	−60.2 (3)	C17—C18—C19—C22	170.9 (5)
Cl2—Cu1—N4—C1	−150.1 (3)	C18—C19—C20—C21	1.9 (8)
N3—Cu1—N4—C5	−160.5 (6)	C22—C19—C20—C21	−172.4 (4)
Cl1—Cu1—N4—C5	−78.8 (4)	C17—N3—C21—C20	−1.8 (7)
Cl2^i^—Cu1—N4—C5	122.6 (4)	Cu1—N3—C21—C20	175.6 (4)
Cl2—Cu1—N4—C5	32.7 (4)	C19—C20—C21—N3	0.7 (8)
C5—N4—C1—C2	0.5 (7)	C23—N2—C22—O1	5.3 (9)
Cu1—N4—C1—C2	−176.9 (4)	C23—N2—C22—C19	−172.0 (5)
N4—C1—C2—C3	−1.8 (8)	C18—C19—C22—O1	−127.1 (6)
C1—C2—C3—C4	1.0 (7)	C20—C19—C22—O1	46.9 (8)
C1—C2—C3—C6	178.9 (4)	C18—C19—C22—N2	50.4 (7)
C2—C3—C4—C5	0.8 (7)	C20—C19—C22—N2	−135.6 (5)
C6—C3—C4—C5	−176.8 (4)	C22—N2—C23—C26	61.0 (6)
C1—N4—C5—C4	1.4 (7)	C22—N2—C23—C25	−179.7 (5)
Cu1—N4—C5—C4	178.6 (3)	C22—N2—C23—C24	−61.0 (6)
C3—C4—C5—N4	−2.1 (7)	N2—C23—C24—C30	−179.4 (4)
C7—N1—C6—O2	0.7 (7)	C26—C23—C24—C30	58.8 (5)
C7—N1—C6—C3	177.9 (4)	C25—C23—C24—C30	−61.0 (5)
C4—C3—C6—O2	162.0 (5)	N2—C23—C25—C28	−179.4 (4)
C2—C3—C6—O2	−15.7 (6)	C26—C23—C25—C28	−59.8 (5)
C4—C3—C6—N1	−15.3 (7)	C24—C23—C25—C28	60.5 (5)
C2—C3—C6—N1	167.1 (4)	N2—C23—C26—C32	178.0 (4)
C6—N1—C7—C10	65.9 (5)	C25—C23—C26—C32	59.6 (5)
C6—N1—C7—C8	−174.3 (4)	C24—C23—C26—C32	−59.6 (5)
C6—N1—C7—C9	−56.5 (6)	C23—C25—C28—C29	−59.7 (5)
N1—C7—C8—C12	179.5 (4)	C23—C25—C28—C27	60.0 (5)
C10—C7—C8—C12	−59.6 (5)	C32—C27—C28—C29	59.7 (5)
C9—C7—C8—C12	60.0 (5)	C32—C27—C28—C25	−60.5 (5)
N1—C7—C9—C14	−178.4 (4)	C25—C28—C29—C30	58.4 (5)
C10—C7—C9—C14	58.4 (5)	C27—C28—C29—C30	−61.1 (5)
C8—C7—C9—C14	−61.0 (5)	C28—C29—C30—C24	−59.1 (6)
N1—C7—C10—C16	178.4 (4)	C28—C29—C30—C31	60.6 (5)
C8—C7—C10—C16	59.8 (5)	C23—C24—C30—C29	60.8 (5)
C9—C7—C10—C16	−58.7 (5)	C23—C24—C30—C31	−58.5 (5)
C7—C8—C12—C13	−60.1 (5)	C29—C30—C31—C32	−59.4 (5)
C7—C8—C12—C11	59.1 (5)	C24—C30—C31—C32	60.5 (5)
C16—C11—C12—C13	60.5 (5)	C30—C31—C32—C27	59.1 (5)
C16—C11—C12—C8	−59.5 (5)	C30—C31—C32—C26	−61.1 (5)
C8—C12—C13—C14	59.0 (5)	C28—C27—C32—C31	−59.1 (5)
C11—C12—C13—C14	−60.3 (5)	C28—C27—C32—C26	60.8 (5)
C7—C9—C14—C15	−58.5 (5)	C23—C26—C32—C31	60.5 (5)
C7—C9—C14—C13	61.4 (5)	C23—C26—C32—C27	−60.4 (5)

Symmetry codes: (i) −*x*, −*y*+1, −*z*+1.

### Hydrogen-bond geometry (Å, °)

**Table:**

*D*—H···*A*	*D*—H	H···*A*	*D*···*A*	*D*—H···*A*
N1—H1A···O1^i^	0.86	2.35	2.969 (5)	129
N2—H2A···Cl1^ii^	0.86	2.66	3.499 (4)	165
O1W—H1WA···O1W^iii^	0.83	2.22	3.00 (4)	159

Symmetry codes: (i) −*x*, −*y*+1, −*z*+1; (ii) −*x*, −*y*+2, −*z*+1; (iii) −*x*+1, −*y*+2, −*z*+1.
